# Characterization of PlyB221 and PlyP32, Two Novel Endolysins Encoded by Phages Preying on the *Bacillus cereus* Group

**DOI:** 10.3390/v12091052

**Published:** 2020-09-21

**Authors:** Audrey Leprince, Manon Nuytten, Annika Gillis, Jacques Mahillon

**Affiliations:** 1 Laboratory of Food and Environmental Microbiology, Earth and Life Institute, UCLouvain, 1348 Louvain-la-Neuve, Belgium; audrey.leprince@uclouvain.be (A.L.); manon.nuytten@uclouvain.be (M.N.); a.gillis@imperial.ac.uk (A.G.); 2 Section of Molecular Microbiology and MRC Centre for Molecular Bacteriology and Infection, Imperial College London, London WC2N 5DU, UK

**Keywords:** bacteriophage, endolysin, *Bacillus cereus*, foodborne pathogen, antimicrobial agent, detection tool

## Abstract

Endolysins are phage-encoded enzymes implicated in the breaching of the bacterial cell wall at the end of the viral cycle. This study focuses on the endolysins of Deep-Blue (PlyB221) and Deep-Purple (PlyP32), two phages preying on the *Bacillus cereus* group. Both enzymes exhibit a typical modular organization with an enzymatically active domain (EAD) located in the N-terminal and a cell wall binding domain (CBD) in the C-terminal part of the protein. In silico analysis indicated that the EAD domains of PlyB221 and PlyP32 are endowed with peptidase and muramidase activities, respectively, whereas in both proteins SH3 domains are involved in the CBD. To evaluate their antimicrobial properties and binding specificity, both endolysins were expressed and purified. PlyB221 and PlyP32 efficiently recognized and lysed all the tested strains from the *B. cereus* group. Biochemical characterization showed that PlyB221 activity was stable under a wide range of pHs (5–9), NaCl concentrations (up to 200 mM), and temperature treatments (up to 50 °C). Although PlyP32 activity was less stable than that of PlyB221, the endolysin displayed high activity at pH 6–7, NaCl concentration up to 100 mM and the temperature treatment up to 45 °C. Overall, PlyB221 and PlyP32 display suitable characteristics for the development of biocontrol and detection tools.

## 1. Introduction

Bacteriophages or phages are viruses that specifically infect bacteria. They are extremely diverse regarding their morphology, genomic material, and lifestyle [1]. The most common phage lifestyles encountered are the lysogenic and lytic cycles. In a lysogenic cycle, after receptor recognition and DNA injection, temperate phages coexist within their host, either integrated in the chromosome or as independent plasmid-like molecules, in a state called prophage [2,3]. During a lytic cycle, after the phage multiplication using the host replication and translation machineries, the virion progeny is released in a lytic process causing the host death. Phage-encoded enzymes, called endolysins, mediate bacterial lysis [4] via peptidoglycan (PG) degrading activity [5]. In general, endolysins do not possess a signal sequence to be transported through the plasmidic membrane. They rather cooperate with another protein, the holin, to achieve cell lysis [6]. At the end of the lytic cycle, endolysins accumulate in the cytoplasm until holins have oligomerized and formed pores in the membrane, allowing endolysins to reach the PG layer, in a tight time regulated process [7,8].

Endolysins encoded by Gram-positive infecting phages display a modular structure with an N-terminal enzymatically-active domain(s) (EAD) and a C-terminal cell wall-binding domain(s) (CBD), both separated by a linker sequence [5]. The EAD contains one or several domains involved in the cleavage of specific bonds in the PG meshwork. These enzymatic activities can be divided into four main classes based on the targeted link or cleavage mechanism: glycosidases (including *N*-acetylglucosaminidase, *N*-acetylmuramidase, and transglycosylases), amidases, endopeptidases, and cysteine, histidine-dependent amidohydrolases/peptidases (CHAP). Less conserved than the EAD, the CBD is responsible for recognition of the host PG and allows keeping the endolysin in close proximity with the cell wall, thus avoiding the release of the enzyme which could lead to unwanted cleavages of neighboring hosts [9]. CBD usually display structural repeats and have been broadly classified into four classes: SH3 domains, choline-binding modules, three-helix bundles, and α/β multimers [5].

Endolysins can be used as antimicrobial compounds in the food and medical sectors instead of their parental phages [10]. Indeed, phages sometimes have such a narrow host spectrum that they cannot target all the pathogenic strains of a certain group whereas their corresponding endolysins usually display a broader spectrum [11]. Moreover, no bacterial resistance to endolysins has been identified so far. This is mainly due to the fact that the rise of such resistant strains would involve important changes in highly conserved PG bonds that are essential for bacterial survival [12]. Endolysin CBDs have also been fused with fluorescent markers or paramagnetic beads and used as specific detection tools [13]. Additionally, endolysins and their CBDs can be engineered (e.g., domain swapping or mutagenesis) to modulate their activity, specificity, or stability [14].

The *Bacillus cereus* group is a highly debated cluster of closely related species. *B. cereus sensu stricto* (s.s), *Bacillus thuringiensis*, *Bacillus anthracis*, *Bacillus weihenstephanensis*, *Bacillus mycoides*, *Bacillus pseudomycoides*, and *Bacillus cytotoxicus* are the seven species most commonly recognized as members of this group, but other species have been suggested as new members [15,16]. In the last few years, several endolysins of phages infecting diverse *B. cereus* group members have been identified and characterized [11,17,18,19,20,21]. Deep-Blue [22,23] and Deep-Purple [24], two phages infecting the *B. cereus* group, belong to the families *Herelleviridae* and *Siphoviridae*, respectively.

The present work focuses on the identification and detailed characterization of the endolysins PlyB221 and PlyP32, encoded by the Deep-Blue and Deep-Purple phages, respectively. Bioinformatic analyses indicated that although related to other endolysins, PlyB221 and PlyP32 display unique SH3 domains as CBD. The activity and binding experiments showed that they were active against members of the *B. cereus* group, as well as some *Bacillus* spp. Overall, the biochemical characterization indicates that both endolysins have properties required for applications in food processing facilities, including activity on a broad range of pH and salt tolerance, and showed stability with respect to temperature treatments.

## 2. Materials and Methods

### 2.1. Bacterial Strains and Growth Conditions

Bacteria were grown overnight (O/N) in Lysogeny Broth (LB) or LB-agar plates at 30 °C for *Bacillus* spp. and at 37 °C for *Escherichia coli* and *Staphylococcus epidermidis*. *Listeria ivanovii* was grown at 37 °C in brain heart infusion (BHI). *E. coli* 10-beta and BL21(DE3) (Millipore corporation, Burlington, MA, USA) competent cells were used for cloning and expression experiments, respectively. *E. coli* cells containing the pET30a vector were selected in LB medium containing 50 µg/mL of kanamycin (Sigma, Saint-Louis, MO, USA) at 37 °C, unless otherwise indicated.

### 2.2. Bioinformatic Analysis

Bioinformatic analyses were done using BLASTp [25], InterPro [26], and HHpred [27]. Protein sequence alignments were done using MUSCLE [28]. The comparative dendrogram was built with MEGA7 [29] using the maximum likelihood method. Protein sequences of phage endolysins were retrieved from NCBI: PlyB221 - YP_009285532.1, PlyP32- ARW58283.1, PlyL - WP_000405763.1, B4 (LysB4) - YP_006908235.1, Phrodo (PlyP56) - YP_009289935.1, BtCS33 (PlyBt33) - YP_006488688.1, PBC5 (LysPBC5) - AKQ08598.1, Bcp1 (PlyB) - YP_009031336.1, PlyPH - WP_000540633.1, BigBertha - YP_008771084.1, TsarBomba - ARW58283.1, Nigalana - YP_009282466.1, PBC1 - AFE86261.1, PBC2 - AKQ08512.1, BPS13 - YP_006907567.1, vB_BceM-HSE3 - AWD93052.1, vB_BthS_BMBphi - AXF39889.1, AP50 - YP_002302543.1, 12,826 - CAA72264.1, TP21-L - CAA72267.1, Bastille - AEQ34462.1, Gamma - YP_338200.1, PBC4 - AKQ08217.1.

### 2.3. Cloning Experiments

The endolysin genes, *gpB221* and *gpP32,* were cloned into the expression vector pET30a (Millipore corporation) after PCR amplification with the Q5 polymerase (NEB) followed by restriction (enzymes from NEB) and ligation with T4 ligase (Promega, Madison, WI, USA) to generate pET30a::*gp221* and pET30a::*gp32*, respectively (Table 1). Constructions were transformed in chemically competent *E. coli* 10-beta, verified by Sanger sequencing (Macrogen, Amsterdam-Zuidoost, The Netherlands) and transformed into *E. coli* expression strain BL21(DE3). Similarly, the regions encoding PlyB221 CBD (residues 149–313) and PlyP32 CBD (181–262) were cloned into pET30a using the protocol described above. An N-terminal GFP tag was then subcloned by restriction-ligation. A linker composed of seven alternating glycine and serine residues was added between the GFP and the CBD. The final recombinant plasmids were named pET30a::*gfp*(linker)::*gp221_cbd* and pET30a::*gfp*(linker)::*gp32_cdb*. Bacteria and plasmids used in cloning and expression experiments are described in Table 1. Sequences of the primers used are listed in Table 2.

### 2.4. Protein Expression and Purification

LB broth was inoculated with *E. coli* BL21(DE3) containing the recombinant vectors and incubated O/N with an agitation of 180 rpm. O/N cultures were diluted 1:10 in fresh LB broth, incubated until the OD_600_ reached 0.5–0.8 and induced with 1 mM Isopropyl β-D-1-thiogalactopyranoside (IPTG) (Sigma). *E. coli* cultures were incubated during 6 h at 28 °C and 180 rpm to allow protein expression. The cells were then centrifuged (4000× *g*, 4 °C for 15 min) and the pellet stored at −20 °C. The pellet was thawed on ice for 30 min and resuspended in lysis buffer (50 mM NaH_2_PO_4_, 300 mM NaCl, 10 mM imidazole pH 8) supplemented with a protease inhibitor cocktail (1x, SIGMAFAT^TM^ Protease Inhibitor Cocktail Tablet, EDTA-Free, Sigma) and 1 mg/mL lysozyme (Sigma). After incubation for 30 min at 10 °C to lyse the cells, three units/mL of Benzonase Nuclease (Sigma) were added to reduce viscosity (15 min incubation). The lysate was centrifuged (30 min, 4 °C at 10,000× *g*) and the supernatant, containing the soluble proteins, was filtered on 0.45 µm (VWR, Oud-Heverlee, Belgium). The recombinant proteins were then purified on Ni-NTA column (Qiagen, Hilden, Germany) according to the manufacturer’s recommendations. The purity was assessed by SDS-PAGE and the recombinant proteins were dialyzed O/N with agitation against PBS supplemented with 300 mM NaCl. The protein concentration was determined by the Bradford assay [30].

### 2.5. Spot-on-Plate Assay

The phage host spectra were assessed by spot-on-plate. Briefly, 5 mL of top agar (LB 0.5% agar, 3 mM CaCl_2_, 3 mM MgCl_2_) were inoculated with 100 µL of an O/N bacterial culture and poured onto a LB-agar plate. After solidification, 10-fold serial dilutions of phage suspensions were spotted on plates containing the bacteria and incubated at 30 °C O/N.

### 2.6. Endolysin Activity Spectrum

The lytic activity of the two endolysins was tested using a turbidity reduction assay [9] which consists in measuring the decrease in OD_595_ caused by the enzymatic activity. Briefly, an O/N bacterial culture was diluted 100-fold in fresh medium and incubated until mid-log phase (3 to 4 h). After centrifugation (6000× *g*, 5 min) the pellet was washed with PBS and then resuspended in 50 mM Tris-HCl pH 8 (PlyB221) or pH 7 (PlyP32) to reach an OD_595_ between 0.8–1. From this bacterial suspension, 180 µL were transferred to each well of a 96-well plate and mixed with phage endolysins at given concentrations. The plate was incubated at 30 °C in a Multiskan^TM^ FC Microplate spectrophotometer (Thermo Fisher Scientific, Watham, MA, USA) where the OD_595_ was monitored every min for 30 min. Wells containing bacterial suspensions and the protein buffer served as control which was subtracted from the experimental data to obtain the final results, expressed as inhibition percentages. The dose response experiment was performed on *B. cereus* ATCC 10,987 using endolysin concentrations ranging from 3 to 100 µg/mL. For the activity spectrum assays, 100 µg/mL of endolysin were tested on strains described in Table 3.

### 2.7. Endolysin Activity on Purified Cell Wall

Cell wall preparations were obtained following [31] with minor modifications. In brief, *B. cereus* ATCC 10,987 from a fresh plate was used to inoculate 1 L of LB broth. The culture was incubated at 37 °C and 180 rpm until the OD_600_ reached 1. The culture was then centrifuged at 4000× *g*, 4 °C for 20 min and the supernatant was resuspended in 40 mL of cold PBS. The suspension was disrupted by sonication (on ice) and then centrifuged at low speed (1500× *g*, 10 min, 4 °C) to remove unbroken cells before high-speed centrifugation (21,000× *g*, 5 min, 4 °C). The pellet was resuspended in 20 mL of SDS (4%), boiled for 20 min and the suspension was centrifuged (21,000× *g*, 5 min, 4 °C). The pellet was then washed three times with warm (45 °C) deionized water and twice with 1 M NaCl (centrifugation at 21,000× *g*, 5 min, room temperature (RT)). The final steps consisted of four washes with deionized water including a first centrifugation at low speed (1500× *g*, 5 min, RT), followed by high-speed centrifugation (21,000× *g*, 5 min, RT). The final pellet was resuspended in 5–10 mL of Milli-Q water.

To assess the endolysin activity on the bacterial cell wall, the cell wall extracts were diluted to reach an OD_595_ of 0.8–1 and mixed with purified PlyB221 and PlyP32 endolysins at different concentrations in a 96-well plate. The reduction in OD_540_ was monitored every min for 30 min at 30 °C with background shaking using a Multiskan^TM^ FC Microplate spectrophotometer (Thermo Fisher Scientific, Watham, MA, USA).

### 2.8. Endolysin Biochemical Characterization

The biochemical characterization of the PlyB221 and PlyP32 endolysins was done on *B. cereus* ATCC 10,987 using the turbidity reduction assay and 50 µg/mL of endolysin. The temperature stability was evaluated by incubating each endolysin at various temperatures during 30 min and then transferring on ice for 5 min. The effects of pH and NaCl were tested by resuspending the bacteria in different buffers (50 mM citrate buffer for pH 4–5, 50 mM phosphate buffer for pH 6–7, 50 mM Tris-HCl buffer for pH 6–9, 50 mM carbonate buffer for pH 10) and in 50 mM Tris buffer pH 8 (PlyB221) or pH 7 (PlyP32) with increasing amount of NaCl (0–500 mM). The results were expressed as a ratio of the maximal activity. Statistical data were derived from Student test and a *p*-value inferior to 0.05 was considered as significant.

### 2.9. Cell Wall Decoration Assay

Binding of the CBD to the bacterial cells was assessed by a cell wall decoration assay [32]. Briefly, 500 µL of exponentially growing cells (3–4 h) were collected by centrifugation (10,000× *g*, 1 min, 4 °C), washed twice with cold PBS and resuspended in 100 µL of PBS. Five to 10 µg of purified GFP fused CBD were mixed with the bacterial suspension and incubated 15 min at RT with agitation (120 rpm). Control reactions used the protein buffer and purified GFP only. Cells were then centrifuged (10,000× *g*, 1 min, 4 °C) and kept on ice during the three washing steps with cold PBS. Bacteria were then observed under the epifluorescent microscope (Leica AF6000) using a filter with an excitation wavelength ranging from 460 to 500 nm and an exposure time of 400 ms. Images were obtained using the Leica LAS AF software.

## 3. Results

### 3.1. PlyB221 and PlyP32 Display the Canonical Features Of Endolysins

**Figure 1:**
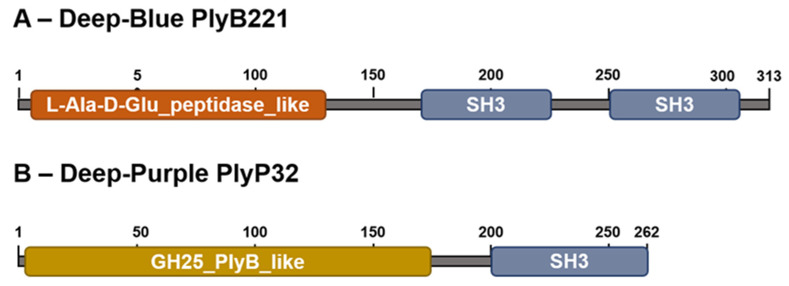
In silico analysis of Deep-Blue PlyB221 and Deep-Purple PlyP32. (**A**) PlyB221 two domain organization. The EAD is a putative L-Ala-D-Glu peptidase domain (superfamily Peptidase_M15) and the CBD contains two potential SH3 domains (superfamily SH3). (**B**) PlyP32 displays a GH25_PlyB_like domain as EAD (GH25 superfamily) characteristic of a muramidase activity and a single SH3 domain as CBD.

**Figure 2:**
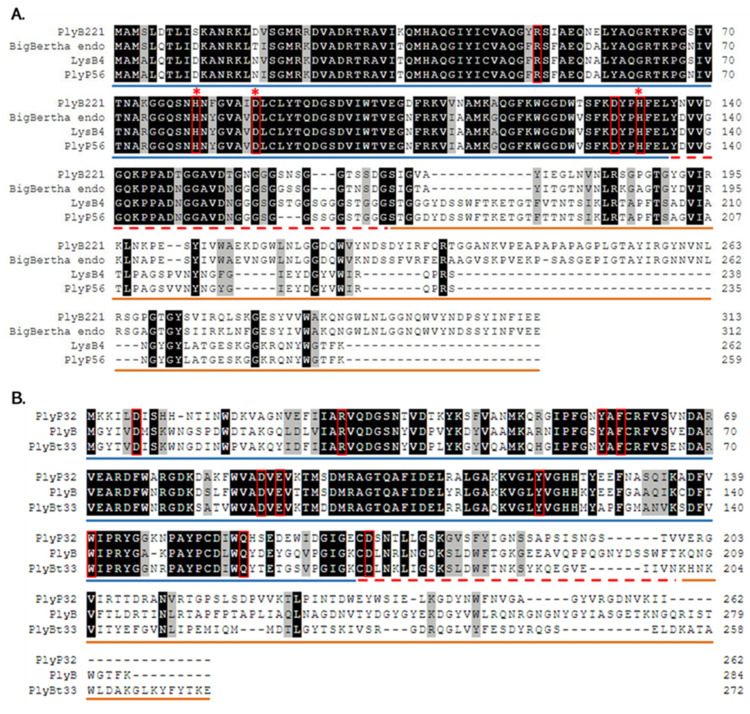
Multiple sequence alignment of PlyB221, PlyP32, and related endolysins. (**A**) Multiple sequence alignment of PlyB221 with endolysins from phages BigBertha, B4 (LysB4), and Phrodo (PlyP56). (**B**) Multiple sequence alignment of PlyP32 with endolysins from phages Bcp1 (PlyB) and BtCS33 (PlyBt33). Residues considered as participating to the active sites are highlighted by red frames, while red stars indicate the Zn^2+^ binding sites in PlyB221. The putative EAD region is underlined in blue, the ‘linker’ sequence with a dashed red line and the CBD in orange.

**Figure 3:**
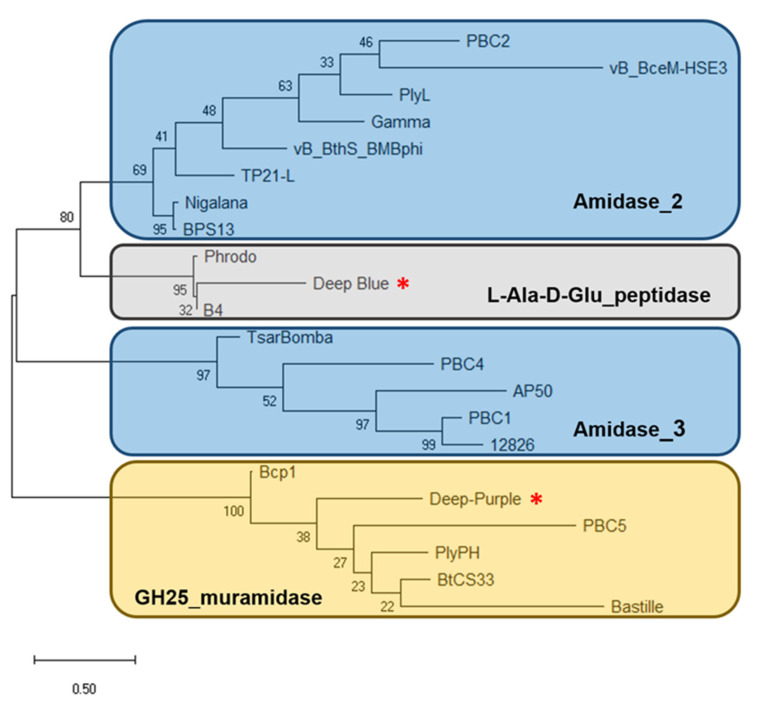
Dendrogram of relationships between the characterized endolysins of *B. cereus* phages. The tree was built using the maximum likelihood method and JTT matrix-based model with 100 bootstrap iterations. Endolysins are indicated by the name of the phage they derived from. Endolysins with the same EAD are grouped in the same cluster. Muramidases, amidases, and endopeptidases are highlighted as yellow, blue, and gray rectangles, respectively. Deep-Blue and Deep-Purple endolysins are indicated by red asterisks. The bar indicates 0.5 substitutions per amino acid site. The numbers refer to the percentage of trees in which the associated endolysins clustered together.

Analysis of phage Deep-Blue genome revealed that gene product (gp) 221 possesses the common organization of phage endolysins (Figure 1A). The gp of this 942-bp gene, renamed PlyB221, indeed displays a putative N-terminal L-Ala-D-Glu_peptidase_like EAD domain (accession number, cd14845, residues 12–135) belonging to the Peptidase_M15 superfamily (cd14814). This suggests that the cleavage site occurs between the alanine and glutamine residues of the peptide chain. PlyB221 presents five potential active sites (Arg50, His80, Asp87, Asp129, His132) of which three (His80, Asp87, His132) are involved in Zn^2+^ binding (Figure 2A). The C-terminal CBD of PlyB221 contains two putative SH3b domains (residues 171–225 and 251–305) from the SH3 superfamily (smart00287) which was confirmed by HHpred analysis that detected two regions (residues 173–230 and 241–304) of structural homologies with the SH3 domains of phage PBC5 endolysin targeting *B. cereus* (Probability: 97%, Protein data bank (PDB) ID: 6ILU_B) [33]. BLAST analysis also showed that PlyB221 is closely related to phage BigBertha endolysin (YP_008771084) as they share 80% identity with 100% coverage (Figure 2A). Besides, this protein is the only one that matches PlyB221 with a high coverage as the other endolysins only cover 48% of PlyB221 sequence, corresponding to its EAD. PlyB221 CBD thus seems to be highly variable and different from the other SH3b domains identified so far. The in silico analysis and protein sequence alignments also revealed that PlyB221 EAD is closely related to that of LysB4 (88% identity) and PlyP56 (85% identity), the endopeptidases of *B. cereus* phages B4 and Phrodo, respectively (Figure 2A) [18,20]. This was confirmed through a comparison gathering all the characterized endolysins encoded by *B. cereus* phages and showing that PlyB221 clusters with LysB4 and PlyP56 (Figure 3).

Regarding phage Deep-Purple, gp32 (789 bp) encodes the endolysin PlyP32 (Figure 1B) with a putative N-terminal GH25_PlyB_like domain (cd06523, residues 3–173) as EAD, which is part of the GH25_muramidase superfamily (cd00599) (i.e., involved in glycosidic bonds cleavage). The 10 potential active sites are conserved in PlyP32 (Asp6, Arg28, Tyr57, Phe59, Asp89, Glu91, Tyr120, Trp140, Gln157, Asp171) and no metal binding site was detected (Figure 2B). One putative SH3b domain was detected using InterPro (G3DSA:2.30.30.40, residues 200–262). Similarly, HHpred highlighted structural homologies with the SH3b domains of endolysins LysF1 from the *Staphylococcus* phage K (Prob. 91%, PDB ID: 5O1Q_A) and LysPBC5 of the *Bacillus* phage PBC5 (Prob. 96%, PDB ID: 6ILU_B) as well as other phage and bacteria-related SH3 domains [33,34]. BLAST analysis indicated that PlyP32 is related to PlyB (coverage 86%, identity 59%) and PlyBt33 (coverage 71%, identity 67%) from *Bacillus* phages Bcp1 and BtCS33, respectively (Figure 3) [19,35]. Similarly to PlyB221, PlyP32 N-terminal part is far more conserved than its C-terminal end (Figure 2B) as indicated by the BLAST analysis that displayed only relevant matches for the EAD domain (i.e., less than 77% coverage).

### 3.2. PlyB221 and PlyP32 Show a Bacteriolytic Spectrum Broader than That of Their Parental Phages

**Figure 4:**
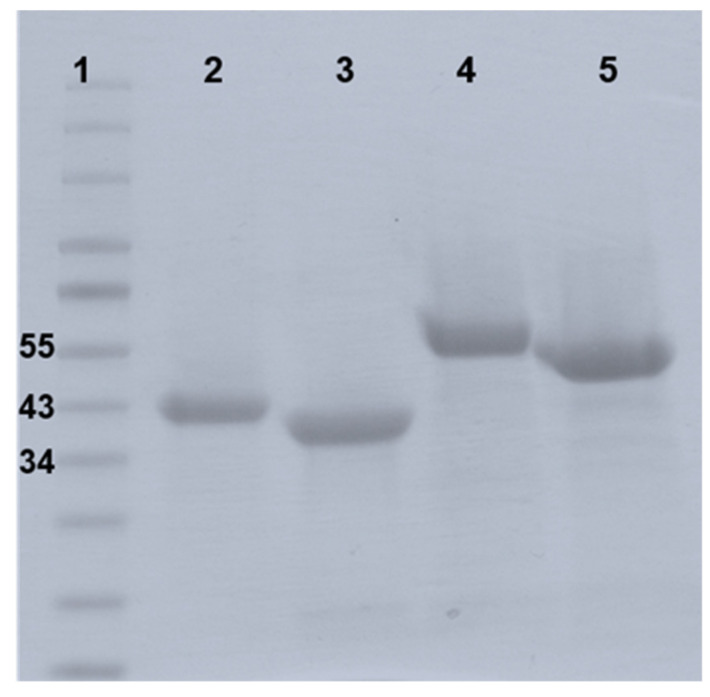
SDS-page gel of purified endolysins PlyB221 and PlyP32. The recombinant proteins were expressed in *E. coli* BL21(DE3) and purified thanks to a six-histidine tag on a Ni-NTA column. Lane 1: Protein ladder in kDa; Lane 2: PlyB221; Lane 3: PlyP32; Lane 4: GFP::PlyB221_CBD; Lane 5: GFP::PlyP32_CBD.

The full *plyB221* and *plyP32* genes were cloned in the expression vector pET30, expressed in *E. coli* BL21(DE3) and purified on a Ni-NTA affinity column to characterize the bacteriolytic activity of the corresponding enzymes. Figure 4 shows the purified recombinant proteins displaying the expected MW of 39 (lane 2) and 34 kDa (lane 3) for PlyB221 and PlyP32, respectively.

**Figure 5:**
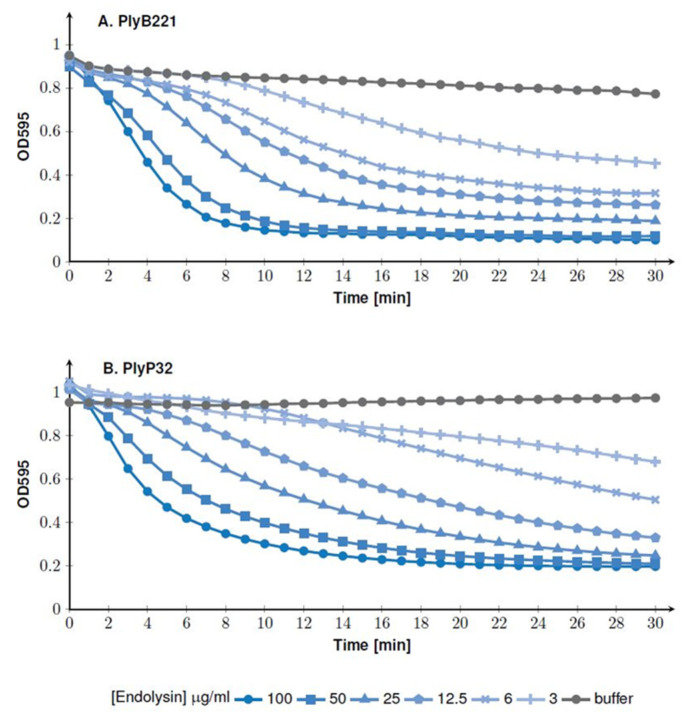
Bacteriolytic activity of PlyB221 (**A**) and PlyP32 (**B**) assessed by OD monitoring. The activity test was performed on exponentially growing *B. cereus* ATCC 10,987 at 30 °C in the optimal buffer for each endolysin and using enzyme concentrations ranging from 3 to 100 µg/mL.

The lytic activity was assessed in a turbidity reduction assay using *B. cereus* ATCC 10,987 as model strain. The endolysins were serially diluted (from 100 to 3 µg/mL) and incubated with the bacteria resuspended in a Tris-HCl buffer (pH 8 for PlyB221 and pH 7 for PlyP32, see below). The test was performed at 30 °C and the OD was monitored during 30 min. Figure 5A shows that at the highest concentration (i.e., 100 µg/mL), PlyB221 induced a 50% reduction in OD within 4 min of incubation. The same reduction was observed after 15 min at an endolysin concentration of 6 µg/mL. The same dose response behavior was observed for PlyP32 and 50% of inhibition was reached within ca. 5 min at a 100 µg/mL protein concentration (Figure 5B).

The activity ranges of PlyB221 and PlyP32 were then established by subjecting strains from the *B. cereus* group (*n* = 14) and other Gram-positive bacteria (*n* = 8) to 100 µg/mL of protein in a turbidity reduction assay. In parallel, the susceptibility of these strains to the parental phages, Deep-Blue and Deep-Purple, was assessed via spot-on-plate. Three types of bacterial responses were recorded (Table 3): sensitive strains (i.e., formation of phage plaques), insensitive strains and those affected by the process of ‘lysis from without’—i.e., no individual plaques during spot assay—but nonetheless cell lysis when phages were applied at a high multiplicity of infection (MOI) [36].

As shown in Table 3, all the tested strains inside the *B. cereus* group were sensitive to both endolysins, although to different extents, ranging from 47% to 76% of inhibition for PlyB221 and from 15% to 79% for PlyP32. Interestingly, phages Deep-Blue and Deep-Purple themselves displayed narrower host spectra as they infected three and four strains, respectively, out of the 14 tested strains of the *B. cereus* group. Thus, the PlyB221 and PlyP32 endolysin activity spectra are much broader than those of their parental phages.

Outside the *B. cereus* group, none of the tested strains were sensitive to the phages, albeit PlyB221 and PlyP32 were able to lyse *Bacillus megaterium*, *Bacillus licheniformis*, and *Bacillus pumilus*. PlyB221 was highly active against *B. megaterium* (i.e., 74% and 80% of inhibition for strains Si0003 and 10A-B3, respectively) but showed reduced activity for *B. licheniformis* and *B. pumilus* compared to members of the *B. cereus* group (i.e., 23%, 7%, and 28% for strains HT00023, Si0279, and 10A-B5 respectively). Concerning PlyP32, the percentage of inhibition detected against the strains of *B. megaterium*, *B. licheniformis*, and *B. pumilus* were around 35%, 40%, and 25%, respectively. No endolysin activity was detected on *Bacillus subtilis*, *L. ivanovii*, or *S. epidermidis* (Table 3).

### 3.3. PlyB221 and PlyP32 Display a Peptidoglycan Hydrolyzing Activity

**Figure 6:**
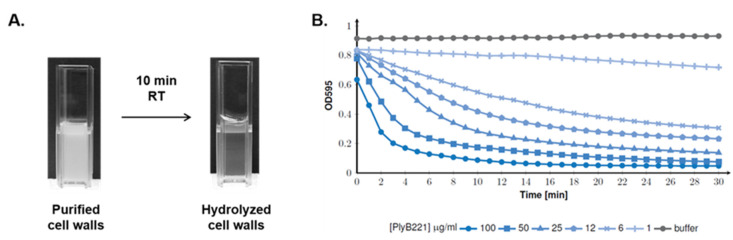
PlyB221 activity on purified cell wall extracts. Cell wall extracts from *B. cereus* ATCC 10,987 were purified and mixed by PlyB221. (**A**) Macroscopic observation *of B. cereus* cell wall hydrolysis with 100 µg of endolysin after 10 min at RT. (**B**) OD_595_ monitoring during 30 min (30 °C) of cell wall hydrolysis using endolysin concentrations ranging from 1 to 100 µg/mL.

To assess the endolysins PG hydrolyzing activity, cell walls form *B. cereus* ATCC 10,987 were purified. After 10 min incubation with the PlyB221 (100 µg/mL), PG hydrolysis can be observed macroscopically (Figure 6A). This was confirmed by OD_595_ monitoring during 30 min as the maximal enzymatic activity of the endolysin is displayed during the first five min (Figure 6B). As for the effect of the endolysin on bacterial cells (Figure 5), PlyB221 efficacy increased with concentration (Figure 6B). Similar observations were made for PlyP32 (data not shown).

### 3.4. PlyB221 Displays a Somewhat more Stable Activity than PlyP32

**Figure 7:**
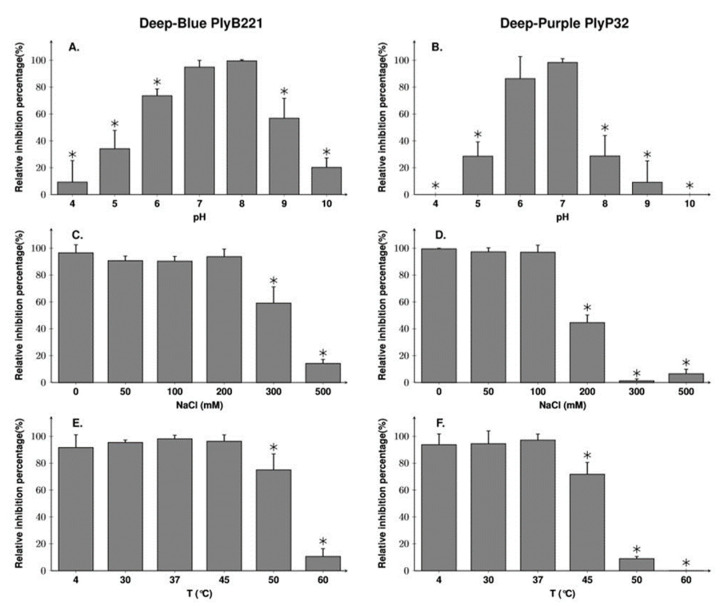
Biochemical characterization of PlyB221 and PlyP32. Exponentially growing *B. cereus* ATCC 10,987 cells were resuspended in buffer of various pH (**A**,**B**) and NaCl concentration (**C**,**D**) and challenged with 50 µg/mL of protein during 30 min to assess the endolysins optimal activity. PlyB221 and PlyP32 stability to temperature (**E**,**F**) was tested by incubating the proteins (50 µg/mL) at given temperatures during 30 min and transferred for 5 min on ice. The experiments were done in triplicate. Stars indicate significant differences (Student’s *t*-test, *p* < 0.05).

The optimal pH conditions and NaCl concentrations for the activity of each endolysin were then established using *B. cereus* ATCC 1087 in a turbidity reduction assay. PlyB221 was active in a wide range of pH (5–9) but the highest activity was obtained at pH 7 and 8 (Figure 7A). PlyP32 optimum was between pH 6 and 7 but, outside this range, its activity dropped to 30% at pH 5 and 8 and no activity was detected at pH 4 or 10 (Figure 7B). For both endolysins, the pH optima determined experimentally are consistent with their predicted p.I, namely 7.67 for PlyB221 and 6.22 for PlyP32. Regarding tolerance to NaCl (evaluated at the endolysin optimal pH, i.e., pH 8 and pH 7 for PlyB221 and PlyP32, respectively), PlyB221 and PlyP32 activities decreased at salt concentrations higher than 300 and 200 mM, respectively (Figure 7C,D). Finally, the endolysin thermal stability was assessed by incubating the proteins at temperatures from 4 to 60 °C during 30 min followed by a 5 min incubation on ice before assessing the residual activity on *B. cereus* ATCC 10987. PlyB221 maximal enzymatic activity was preserved up to 45 °C and the protein retained 75% activity after 50 °C (Figure 7E). PlyP32 enzyme was stable up to 37 °C and lost 28% activity at 45 °C. After 50 °C, the enzymatic activity dropped to 9% and was completely inhibited after incubation at 60 °C (Figure 7F).

### 3.5. PlyB221 and PlyP32 Cell Wall Binding Domain (CBD) Can Decorate Bacillus Cells

**Figure 8:**
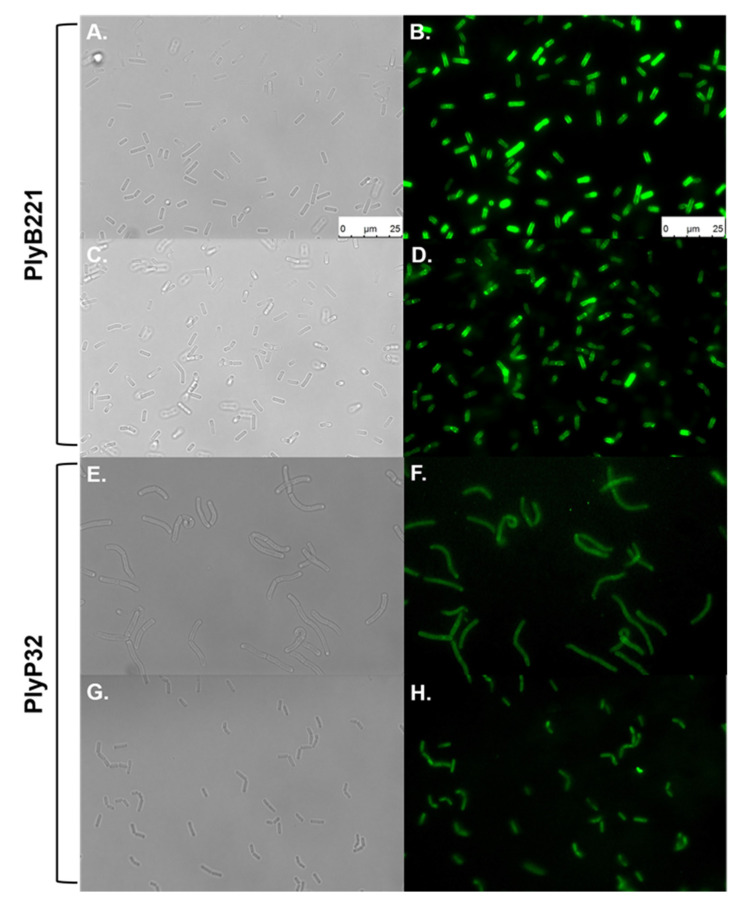
Binding ability of PlyB221 and PlyP32 CBD. Exponentially growing cultures were used in a cell wall decoration assay based on the observation of the GFP fused CBD adsorbed to bacteria by fluorescent microscopy. Images on the left show bright field microscopy while images on the right display the corresponding fluorescent images. (**A**,**B**) *B. cereus* ATCC 10,987 - PlyB221_CBD; (**C**,**D**). *B. weihenstephanensis* Si0239 - PlyB221_CBD; (**E**,**F**) *B. megaterium* Si0003 - PlyP32_CBD; (**G**,**H**) *B. weihenstephanensis* LH002 - PlyP32_CBD. Scale bar = 25 µm for all panels.

The role of the C-terminal SH3b domains as CBD was evaluated in a cell wall decoration assay, which relies on the observation, by fluorescent microscopy, of the green fluorescent protein (GFP) fused to CBD and adsorbed onto bacteria. N-terminal GFP fusions of the endolysin CBDs (residues 149 to 313 for PlyB221_CBD and 181 to 262 for PlyP32_CBD) (Figure 1) were cloned and expressed in *E. coli* BL21(DE3). The purified recombinant proteins are shown on Figure 4 with their MW corresponding to 50 kDa for GFP::PlyB221_CBD (lane 4) and 42 kDa for GFP::PlyP32_CBD (lane 5). Figure 8 shows that the CBD of both PlyB221 and PlyP32 decorate uniformly the bacteria confirming the role of the SH3 domain as recognition modules. The purified GFP tag alone did not adsorb to the bacteria (data not shown). All the strains belonging to the *B. cereus* group were recognized, regardless of their sensitivity towards phages, thus showing that the endolysin binding spectra are wider (Table 3). Overall, the fluorescence observed with PlyB221_CBD was more intense than that recorded with PlyP32_CBD (Figure 8B–H). Outside of the *B. cereus* group, the other assayed Gram-positive bacteria were not recognized apart from the two *B. megaterium* strains (Table 3, Figure 8F).

## 4. Discussion

Endolysins are phage-encoded enzymes that mediate the bacterial lysis during the last step of the viral lytic cycle. Thanks to their EAD enzymatic properties and their CBD allowing recognition and binding to the host cell, endolysins have gained much attention as alternative antimicrobials and detection tools for pathogenic bacteria [46]. In this study, we focused on PlyB221 and PlyP32 endolysins, encoded by two phages preying on the *B. cereus* group. Deep-Blue is a lytic phage belonging to the *Herelleviridae* whose potential for biocontrol purposes was previously investigated [23]. It was able to efficiently reduce the contamination by emetic *B. weihenstephanensis* in food matrices and biofilms, but its main drawback remained its narrow host range, therefore indicating that it should be included in a phage cocktail. Similarly, the siphovirus Deep-Purple was only able to infect a limited number of strains belonging to the *B. cereus* group but in addition, it was hypothesized that it might be a lytic variant of a lysogenic phage, making it unsuitable for biocontrol purposes [24]. To get around these drawbacks, their respective endolysins were investigated in this study.

As for other endolysins encoded by phages infecting Gram-positive bacteria, PlyB221 and PlyP32 have an EAD located at their N-terminal end. BLAST and phylogenetic analysis of PlyB221 indicated that it is close to LysB4 and PlyP56 encoded by *B. cereus* phages B4 and Phrodo, respectively. Similarly, its enzymatic function is fulfilled by an L-Ala-D-Glu_peptidase domain that is predicted to have three metal-binding sites. It was previously shown for LysB4 and PlyP56, that the activity is lost in the presence of EDTA and then restored with the addition of metal ions—such as zinc, calcium, and manganese—thus reinforcing the fact that PlyB221 also relies on divalent cations for its activity [18,20]. PlyP32 possesses a GH25_muramidase EAD that is similar to that of PlyB, the endolysin encoded by the *Bacillus* phage Bcp1, and PlyBt33 from phage BtCS33 [47,48]. Both PlyB221 and PlyP32 displayed high lytic activity within the first 10 min of incubation with the most sensitive bacteria when 100 µg/mL of protein was tested, which corresponds to 2.6 and 2.9 µM, respectively. This antimicrobial activity is similar to what has been previously described for endolysins targeting the *B. cereus* group.

Endolysins encoded by phages infecting the *B. cereus* group show differences regarding the targeted bacteria, ranging from a narrow to a broad activity spectrum. For instance, LysPBC5 is only able to lyse members of the *B. cereus* group whereas LysPBC2 is also active against other *Bacillus* spp., and even *L. monocytogenes* and *Clostridium perfringens* [11,33]. PlyB221 and PlyP32 both have intermediate activity spectra, as they are active against strains of the *B. cereus* group but also *B. megaterium*, *B. pumilus*, and *B. licheniformis*. Noteworthy, PlyB221 and PlyP32 were not tested against *B. anthracis*, but based on their close relationships to PlyP56 and PlyB whose activity against *B. anthracis* was demonstrated [20,47], their activity spectrum is expected to include this pathogenic bacterium. Also, PlyB221 and PlyP32 are active on a much wider range of bacteria than that of their parental phages. These broad activity spectra could target most, if not all, of the pathogenic strains of the *B. cereus* group.

Endolysin activity is mainly influenced by pH and ionic strength and those encoded by *B. cereus* phages usually display optimal activity at neutral or slightly basic pH, and tolerate up to 100–200 mM [17,20,35]. PlyB221 showed high activity (>50%) from pH 6 to 9 whereas PlyP32 activity rapidly dropped outside the range of pH 6 and 7. Endolysins from phages targeting *B. cereus* that are effective at pH 6 have already been described (i.e., PlyPH and PlyB) but they were also highly active up to pH 9, contrary to PlyP32 [47,49]. The addition of salt did not improve the activity of PlyP32 and PB221, contrary to what was observed for PlyB, and they were able to withstand up to 100 mM and 200 mM of NaCl, respectively, without any loss of activity. The thermal stability of endolysins is also important for their potential application in the food industry. Both endolysins retained high lytic activity (>50%) after treatments up to 45–50 °C which, although not as resistant as some *Listeria* specific endolysins (e.g., HPL511 70% activity after 30 min at 70 °C [50]) is consistent with other endolysins encoded by phages infecting *B. cereus* [35,51]. Overall, PlyB221 appeared to be active in a wider range of pH and NaCl conditions and withstood higher temperature treatments than PlyP32.

In both endolysins, the CBD is represented by SH3 domains, a type of CBD widespread among endolysins of phages infecting *B. cereus* along with the Amidase02_C domain (PF12123) [17,21,47,52]. Contrary to the endolysins activity range that extended to several species outside the *B. cereus* group, binding of both CBD was limited to strains of the *B. cereus* group and to *B. megaterium* (Table 3). This observation suggests that the motif recognized by the SH3b domain is common to strains belonging to both *B. cereus* and *B. megaterium*. Although it is still unclear what the SH3 domain ligand is, Lee et al. have shown that the CBD of LysPBC5 is composed of tandem SH3 domains that bind to the PG glycan strands rather than to the peptide interbridge as previously thought [33,53]. Interestingly, although both PlyB221 and PlyP32 endolysins display the same binding spectra, the fluorescent bacteria decorated with PlyB221_CBD were always brighter than those recognized by PlyP32_CBD.

## 5. Conclusions

In conclusion, we identified two novel endolysins that showed promising characteristics that open several avenues to develop applications for detecting and bio-controlling *B. cereus*. Beyond their intrinsic binding and activity, their respective EAD and CBD add up to the increasing amount of identified endolysin domains that could be used to synthesize chimeric proteins with improved activity, binding, and stability properties.

## Figures and Tables

**Table 1:** Strains and plasmids used for cloning and expression experiments.

Strains/Plasmids	Species/Purpose	Reference
**Strains**		
10-beta	*E. coli*/cloning strain	NEB
BL21(DE3)	*E. coli*/expression strain	Novagen
**Plasmids**		
pET30a	Expression plasmid	Novagen
pUC18::***gfp***	GFP amplification	Clontech/Takara
pET30::***gp221***	Test PlyB221 lytic activity	This study
pET30::***gfp***(linker)::***gp221_cbd***	Test PlyB221 CBD binding activity	This study
pET30::***gp32***	Test PlyP32 lytic activity	This study
pET30::***gfp***(linker)::***gp32_cdb***	Test PlyP32 CBD binding activity	This study

**Table 2:** Primers used in this study. The extending ends are highlighted in bold.

Target	Primer Name	Sequence (5′ → 3′)
***gfp***	GFP_EcoRI_F	**TTCCGAATTC**AAAGGAGAAGAACTTTTCACTGGAG
	GFP_EagI_linker_R	**TTCGGCCGTCCACTACCTGATCCACTACC**TTTGTAGAGCTCATCCATGCC
***gp221***	gp221_NcoI_F	**TATCCATGG**TGGCAATGTCTTTAGATACTTTAATCA
	gp221_XhoI_R	**TATTCTCGAG**CTACTCCTCAATGAAGTTGATGTATG
***gp221_cbd***	gp221_CBD_EagI_F	**AGCGGCCG**GGTGGAGCAGTAGACACT
	gp221_XhoI_R	**TATTCTCGAG**CTACTCCTCAATGAAGTTGATGTATG
***gp32***	gp32_NcoI_F	**TATCCATGG**ACAAAAAGATTTTAGATATTTCACATCAC
	gp32_XhoI_R	**ATTACTCGAG**TTAAATGATTTTTACGTTATCGCCA
***gp32_cbd***	gp32[181]_EagI_F	**AACGGCCG**GTTAGTTTCTATATTGGTAACTCTT
	gp32_XhoI_R	**ATTACTCGAG**TTAAATGATTTTTACGTTATCGCCA

**Table 3:** Activity and binding spectra of PlyB221 and PlyP32 endolysins and their respective cell wall binding domain (CBD) compared to Deep-Blue and Deep-Purple phages host spectra. Phage host spectra were established via spot-on-plate. The lytic activity was assessed in triplicate by a turbidity reduction assay on exponentially growing cells using 100 µg/mL of endolysin. The binding spectra were established in a cell wall binding as assay using exponentially growing cells and the GFP fused CBD. Stars indicate emetic strains.

			Deep-Blue			Deep-Purple		
Strain	Species	Reference	Φ ^a^	PlyB221 ^b^	CBD ^c^	Φ	PlyP32	CBD
**Strains of the *B. cereus* group**								
VD021	*B. cereus*	[37]	S	74.9 ± 3.9	+	S	77.1 ± 2.4	+
ATCC 10987	*B. cereus*	[38]	I	72.6 ± 2.3	+	I	77.8 ± 9.9	+
F4810/72	B. cereus *	[39]	L	67.8 ± 10.7	+	L	70.3 ± 1.7	+
H3081.97	B. cereus *	[40]	L	57.3 ± 5.9	+	I	32.7 ± 0.7	+
WSBC10202	*B. weihenstephanensis*	[41]	S	61.1 ± 3.4	+	L	71.2 ± 8.4	+
WSBC10204	*B. weihenstephanensis*	[41]	L	76.4 ± 9.1	+	L	68 ± 6.8	+
Si0239	B. weihenstephanensis *	[23]	S	66.6 ± 4	+	L	79.3 ± 2.8	+
LH002	B. weihenstephanensis *	[24]	I	69.3 ± 10.5	+	S	75.7 ± 5.7	+
GBJ001	*B. thuringiensis*	[42]	L	76.2 ± 3	+	L	85 ± 0.4	+
HD4	*B. thuringiensis*	BGSC ^1^	L	38.1 ± 0.9	+	S	73.4 ± 7	+
NVH 391-98	*B. cytotoxicus*	[43]	L	74.2 ± 6	+	L	80.4 ± 4.7	+
BcA20 086	*B. cytotoxicus*	MIAE ^2^	L	46.6 ± 11	+	L	34.4 ± 14.2	+
KBS 2–12	*B. mycoides*	MIAE	L	62.1 ± 2.7	+	S	62 ± 4.2	+
KNC2-18	*B. mycoides*	MIAE	I	66 ± 23.3	+	I	15.4 ± 8.6	+
**Other Gram-positive bacteria**								
Bs168	*B. subtilis*	[44]	I	0.2 ± 0.4	-	I	0 ± 0	-
ATCC 10716	*B. licheniformis*	BBCM ^3^	I	23.2 ± 1	-	I	40.1 ± 4.5	-
SI0279	*B. pumilus*	MIAE	I	6.8 ± 0.9	-	I	21.8 ± 7.7	-
10A-B5	*B. pumilus*	[45]	I	28.3 ± 1.5	-	I	26.3 ± 10.2	-
SI0003	*B. megaterium*	MIAE	L	73.9 ± 2.7	+	I	34.2 ± 6.6	+
10A-B3	*B. megaterium*	[45]	I	79.9 ± 0.5	+	I	35.3 ± 14.3	+
Liv001	*L. ivanovii*	MBLG ^4^	I	0.5 ± 0.9	-	I	0 ± 0	-
ATCC 12228	*S. epidermidis*	BBCM	I	0.1 ± 0.2	-	I	0 ± 0	-

^a^ Phage host spectrum: S: Sensitive/plaque forming strain; I: Insensitive strain; L: Strain affected by “lysis from without”; ^b^ Lytic activity: percentage of inhibition; ^c^ Binding activity: + binding; - no binding; ^1^ BGSC: Bacillus genetic Stock Center, Ohio State University, USA.; ^2^ MIAE: Collection of the Food and Environmental Lab, UCLouvain, Louvain-la-Neuve, Belgium.; ^3^ BBCM: Belgian coordinated collection of microorganisms; ^4^ MBLG: Pôle de Microbiologie Médicale, UCLouvain, Woluwe-Saint-Lambert, Belgium.
